# Supplementary material for: Intelligent ICU for Autonomous Patient Monitoring Using Pervasive Sensing and Deep Learning
Source: Sci Rep. 2019 May 29;9:8020. doi: 10.1038/s41598-019-44004-w (PMC6541714; doi:10.1038/s41598-019-44004-w)
Supplement: Supplementary file 1 — Supplemental Digital Content [file 41598_2019_44004_MOESM1_ESM.pdf]

Intelligent ICU for Autonomous Patient Monitoring Using Pervasive Sensing and Deep Learning

Anis Davoudi[1], Kumar Rohit Malhotra[2], Benjamin Shickel[2], Scott Siegel[1], Seth Williams[3,4], Matthew Ruppert[3,4], Emel Bihorac[3,4], Tezcan Ozrazgat-Baslanti[3,4], Patrick J. Tighe[5], Azra Bihorac[3,4,+], and Parisa Rashidi[1,2,4,+,*]

[1]Department of Biomedical Engineering, University of Florida, Gainesville, FL, 32611, USA
[2]Department of Computer and Information Science and Engineering, University of Florida, Gainesville, FL, 32611, USA
[3]Department of Medicine, University of Florida, Gainesville, FL, 32611, USA
[4]Precision and Intelligent Systems in Medicine (PrismaP), University of Florida, Gainesville, FL, 32611, USA
[5]Department of Anesthesiology, University of Florida, Gainesville, FL, 32611, USA
[*]parisa.rashidi@ufl.edu
[+]these authors contributed equally to this work

Appendix A

Face Detection

To detect individual faces, we extracted seven seconds of still images at 15 fps as training data and used the Joint Face Detection and Alignment using Multi-Task Cascaded Convolutional Network (MTCNN). This framework employs a cascaded architecture with three stages of deep convolutional neural networks (CNN) to predict face and landmark locations in a coarse-to-fine manner. In the first stage, candidate windows possibly containing faces are produced using a fully convolutional network called Proposal Network (P-Net) (Figure S3)[1]. Each candidate window has four coordinates – top left coordinates, height, and width. Ground truth bounding boxes have the same coordinate format as well. The objective function for bounding box regression performed on these candidate windows is the Euclidean loss between the corresponding coordinates of a candidate window and its nearest ground truth bounding box. The objective is to minimize this Euclidean loss, given for a sample x_i as in equation (1).

$$L_i^{box} = ||\hat{y}_i^{box} - y_i^{box}||_2^2 \tag{1}$$

Here,$\hat{y}_i^{box}$ is the output regression coordinate obtained from the network and y_i^{box} is the ground-truth coordinate. After performing bounding box regression, the highly overlapping candidates are merged using non-maximum suppression (NMS)[2]. NMS is performed by sorting the bounding boxes by their score, and greedily selecting the highest scoring boxes and removing the boxes that overlap with the already selected boxes more than a given threshold, 0.7 in the first stage. In the second stage, all candidates selected in the first stage are provided to another convolutional network, Refine Network (R-Net) (Figure S3). R-Net further rejects candidate windows not containing faces, performs bounding box regression, and merges the NMS candidates with a threshold of 0.7. Finally, the Output Network (O-Net) produces the final bounding box (Figure S3). MTCNN is trained for bounding box regression by posing its objective function as a regression problem. While extracting the candidate windows during testing, a window is selected on the basis of the threshold given for Intersection over Union (IoU) score, calculated as in equation (2).

$$IoU_i = \frac{A_i^o}{A_i^u} \tag{2}$$

Here, A_i^o is the area of overlap between the *ith* ground-truth bounding box and the ith detected bounding box, and A_i^u is the area of union between the *ith* ground-truth bounding box and the *ith* detected bounding box. If the IoU_i is above the given threshold for a candidate window, the window is selected for the next stage. The three-stage threshold values used for selecting the candidate windows were 0.6, 0.7 and 0.9 respectively. The face thumbnails obtained from this framework have a size of 160*160 pixels. These thumbnails are provided to the face recognition framework as input.

Face Recognition

FaceNet is a deep CNN model that extracts facial features in terms of 128-D Euclidean (L2) embeddings using a triplet-based loss function[3]. The input to FaceNet model is the set of aligned images obtained from MTCNN. The network is trained such that the squared L2 distances in the embedding space directly correspond to face similarity. These embedding vectors can then

be used as feature vectors for a classification model. We used the FaceNet model pre-trained on a subset of the MS-celeb-1M dataset which includes about 10 million images of 100,000 celebrities[4], which had an accuracy of 0.99 on the Labeled Faces in the Wild (LFW) dataset[5]. The pre-trained model was used to extract features from patient thumbnails and to calculate the corresponding L2 embeddings for our training images. These embeddings are then used to train a linear Support Vector Machine (SVM) for classification, on a fixed number of images (n=100, ~7 seconds of video) of a patient, along with the same number of negative examples of non-patients. The tolerance value for stopping criterion was set to 0.001.

Appendix B

Posture Classification

While our recorded patient frames contain examples of functional status activities such as walking, sitting in bed, or sitting on chair, these activities are interspersed in an imbalanced and sparse manner throughout the video clips. To remedy this problem, besides patient data, we additionally recorded 90 minutes of video containing scripted functional activities performed by nonpatients in the same ICU rooms. Out of the 150,621 video frames, 74,924 frames are scripted, and 75,697 frames are taken based on actual ICU patients' videos. The initial size of each frame was 1680x1050, which was reduced to 368x654 to accommodate the memory. We used a multi-person pose estimation model[6] to localize anatomical key-points of joints and limbs. Most algorithms are single-person estimators[7–9], such that they first detect each person and then estimate the location of joints and limbs. The single-person approach suffers from early commitment problem when multiple people are in close proximity; if an incorrect detection is made initially, there is no point of return as this approach tracks the initial detection. Due to the small size of hospital rooms and the presence of multiple people (patient, doctors, nurses, visitors), we used the multi-person approach[6]. It also allows us to decouple the runtime complexity from the number of people for real-time implementations. The multi-person pose estimation was performed using the real-time multi-person 2D pose estimation with part affinity fields. The part affinity fields are 2D vector fields that contain information about the location and direction of limbs with respect to body joints. Our pose detection model consists of two branches of a sequential prediction process, where one branch detects the locations of joints, and the other branch detects the association of those body joints, as limbs. Both branches consist of Fully Convolutional Neural networks (FCN)[10]. A convolutional network, consisting of first 10 layers of VGG-19[11], is used to generate a set of feature maps **F**. These feature maps are used as input to each branch of the first stage of the model. The first branch outputs a set of detection confidence maps $S^1 = \rho^1(F)$ and the second branch outputs a set of part affinity fields $L^1 = \phi^1(F)$ where S^1 and ϕ^1 are the two branches of CNNs at the first stage. In the following stage, the outputs from the branches in the previous stage and the original image features **F** are combined and provided as inputs to the two branches of the next stage, for further refinement. The confidence maps and part affinity fields for the subsequent stages are calculated as in equation (3) and equation (4), respectively[6].

$$S^t = \rho^t(F, S^{t-1}, L^{t-1}), \forall t \geq 2 \tag{3}$$

$$L^t = \phi^t(F, S^{t-1}, L^{t-1}), \forall t \geq 2 \tag{4}$$

This process is followed for the t stages of the network. We have used three stages of the network in our model.

This model has been pre-trained on the MPII Human Pose dataset for 144,000 iterations. It contains over 40K activities with annotated body joints[12]. The final model provided a state-of-the-art mean average precision of 0.79 on MPII dataset. We used the lengths of body limbs and their relative angles as features for the classification model. We used estimated poses to detect the four functional activities. We got the best results with K-Nearest Neighbors for classification, with Minkowski distance metric and value of K equal to one.

During the poselet detection step, sometimes a few anatomical key-points were not detected. This led to the problem of missing values for some features in the data that were provided to the classification model. Most algorithms are not immune to missing values. Several methods can be used to impute missing values, including mean, median, mode, or amputation via k-nearest neighbors (k-NN)[13]. The K nearest neighbors are found based on the distance with the remaining features between the different samples. Each missing value of a feature was imputed by the weighted average of the same feature of the K nearest neighbors, with a K value of three. The resulting poselets were then used to train and test the classification algorithm on our dataset. We used 80% of our data for training, and 20% for testing. The ICU training data included 74,924 frames from the scripted dataset and 75,697 frames from the actual ICU patients. Test data comprised only actual patient data. The hyper-parameters of the classification algorithms were fine-tuned using GridSearchCV with five-fold cross-validation. Pipeline of posture recognition model is shown in Figure 6.

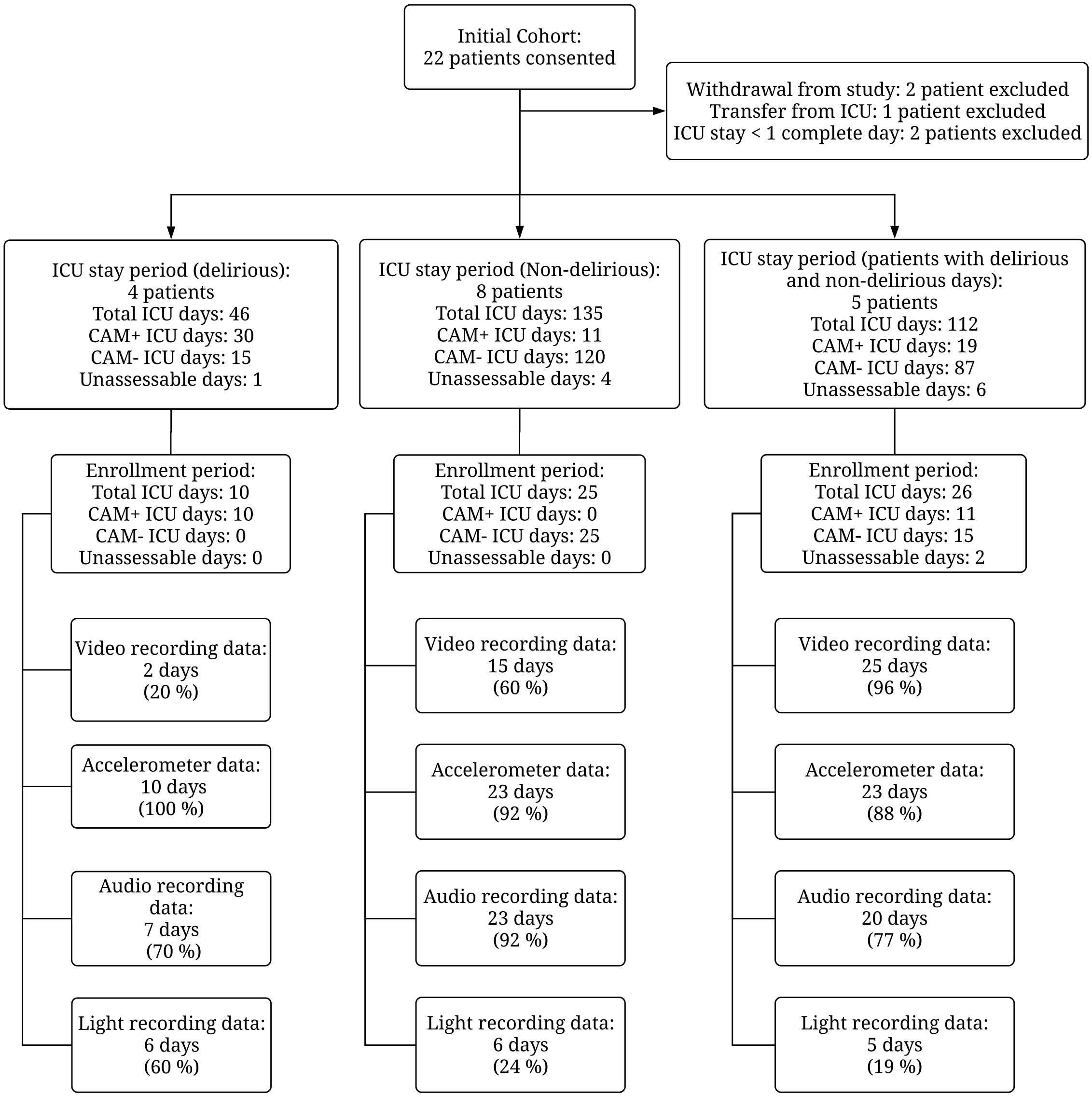

Figure S1. Cohort recruitment diagram. Confusion Assessment Method for the Intensive Care Unit (CAM-ICU) was used to assess patients for delirium. If a patient had any positive CAM-ICU screening for a day, that day was identified as CAM+ (delirious). Days with no positive CAM-ICU screening, but which had negative CAM-ICU screening were identified as CAM- (Non-delirious). Days that the patient could not be assessed because of Richmond Agitation-Sedation Scale score of less than -3 were identified as unassessable days. Patients were divided into three groups: delirious patients (patients who were delirious through their enrollment period), non-delirious patients (patients who were not delirious through their enrollment period), and patients who had both delirious and non-delirious days. CAM: confusion Assessment Method, ICU: Intensive Care Unit.

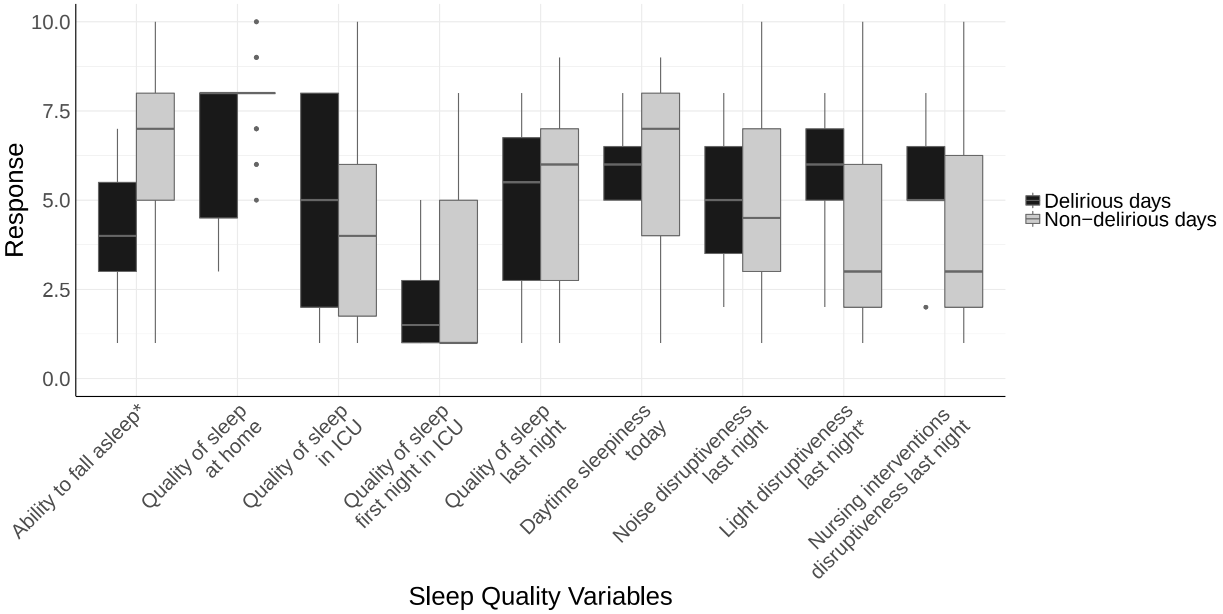

Figure S2. Sleep quality outcomes, patient self-reports using Freedman Sleep Questionnaire. The parameters range from 1 to 10, with 1 being poor and 10 being excellent for the first five criteria. For overall daytime sleepiness, 1 is unable to stay awake, 10 is fully alert and awake. For environment and nursing interventions disruptiveness variables, 1 is no disruption, 10 is significant disruption. *: p-value less than 0.05. Number of delirium nights: 9. Number of non-delirium nights: 43.

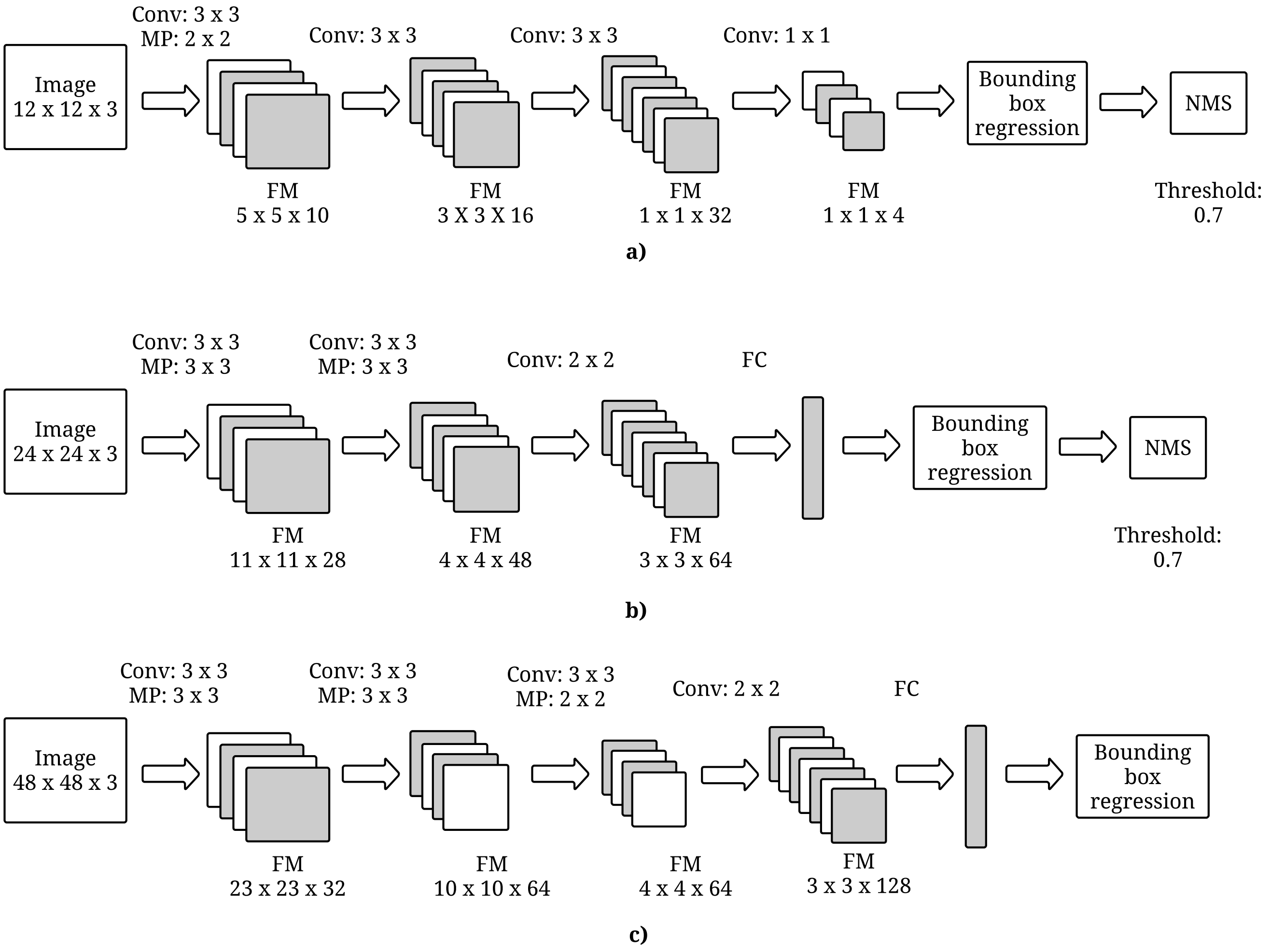

Figure S3. Architecture of face detection network. a) Proposal network (P-Net) produces candidate windows possibly containing faces, b) Refine network (R-Net) rejects candidate windows not containing faces and performs bounding box regression, c) Output network (O-Net) produces the final bounding box. Conv: Convolutional, MP: Max pooling, FC: Fully Connected layer, FM: Feature Maps and NMS: Non-Maximum Suppression. The numbers denote the kernel size in Conv and MP layers. The numbers for FM denote the height, width and depth of the FM. The step-size for each Conv layer is one and for each MP layer is two.

Facial Expression	AUs
Happiness	6+12
Sadness	1+4+15
Surprise	1+2+5+26
Fear	1+2+4+5+7+20+26
Anger	4+5+7+23
Disgust	9+15+16
Contempt	R12A+R14A
Pain	4+6∥7+9∥10+43

Table S1. Action Units (AUs) for each facial expression.

Facial Action Unit Name	Facial Action Unit number	Binary/intensity coding
Inner brow raiser	AU1	Intensity
Outer brow raiser	AU2	Intensity
Brow lowerer	AU4	Intensity/Binary
Upper lip raiser	AU5	Intensity
Cheek raiser	AU6	Intensity
Nose wrinkler	AU9	Intensity
Lip corner puller	AU12	Intensity/Binary
Dimpler	AU14	Intensity
Lip corner depressor	AU15	Intensity/Binary
Chin raiser	AU17	Intensity
Lip stretcher	AU20	Intensity
Lip tightener	AU23	Binary
Lips part	AU25	Intensity
Lip suck	AU28	Binary
Blink	AU45	Binary

Table S2. Action Units (AUs) detected using the OpenFace toolbox.

		Predicted label		
		Lying	Sitting on chair	Standing
True label	Lying	94.45	0.79	4.76
	Sitting on chair	1.73	92.89	5.38
	Standing	4.23	11.97	83.80

Table S3. Confusion matrix showing the model performance for the four postures -lying, sitting in bed, sitting on chair, and standing- using K-Nearest Neighbor model.

Variable, median (IQR)	Non-delirious patient days (N=15)	Delirious patient days (N=3)	p value
Mean activity count 24-hour	25.6 (13.6, 125.9)	4.8 (3.2, 15.2)	0.10
Standard deviation of activity count 24-hour	106.6 (84.5, 346.1)	81.6 (52, 95)	0.20
Mean activity count daytime	33.9 (11.8, 126.9)	6.7 (4.5, 11.9)	0.08
Standard deviation of activity count daytime	139.1 (71.6, 370.1)	54.3 (43, 84.8)	0.08
Mean of activity count nighttime	21.8 (8.9, 66.1)	0 (0, 19.8)	0.15
Standard deviation of activity count nighttime	103.4 (58.1, 296.2)	0 (0 , 70.9)	0.12
M10 [a]	30081.5 (13732.6, 147613.8)	6841.1 (4636.5, 13373.4)	0.06
Time of M10 (hour)	317 (162, 548)	413 (241.5, 545.5)	0.82
L5 [b]	927.5 (593.4, 2789.8)	0 (0, 252.7)	0.04
Time of L5 (hour)	7 (8, 18)	1 (1,4)	0.15
Relative amplitude	0.9 (0.9, 1)	1 (0.97, 1)	0.06
RMSSD [c]	117.6 (103.1, 360.7)	85.6 (56.7, 102.8)	0.20
RMSSD/SD [d]	1.1 (1, 1.2)	1.1 (1.1, 1.2)	0.57
Number of immobile minutes daytime	589 (498.5, 670.5)	683 (636.5, 697)	0.16
Number of immobile minutes nighttime	632 (601.5, 673)	720 (605, 720)	0.29

[a] Activity intensity of 10-hour window with highest sum of activity intensity.
[b] Activity intensity of 5-hour window with lowest sum of activity intensity.
[c] Root Mean Square of Sequential Differences.
[d] Root Mean Square of Sequential Differences/Standard Deviation.

Table S4. Movement features for the arm, comparing between the delirious and non-delirious groups.

Variable, median (IQR)	Non-delirious patient days (N=15)	Delirious patient days (N=6)	p value
Mean activity count 24-hour	8 (7.1, 27.1)	16.6 (8.5, 53.0)	0.46
Standard deviation of activity count 24-hour	61.4 (52.3, 91.2)	57.7 (46.2, 138.8)	0.91
Mean activity count daytime	8.9 (6.6, 28.3)	17.3 (8.7, 57.9)	0.51
Standard deviation of activity count daytime	61.7 (57.2, 98.7)	60.4 (53.9, 130.5)	0.85
Mean of activity count nighttime	9.8 (5.3, 22.4)	22.4 (8.1, 46.8)	0.51
Standard deviation of activity count nighttime	64.6 (42.2, 80.2)	61.8 (32.0, 146.4)	0.91
M10 [a]	8094.1 (6817.2, 27183.4)	18702.3 (10107.9, 44256.8)	0.23
Time of M10 (hour)	6 (2.5, 8.5)	9 (3.2, 13.2)	0.56
L5 [b]	544.4 (287.6, 2067.1)	1226.5 (555.5, 8623.2)	0.29
Time of L5 (hour)	13 (1.5, 16)	10 (5, 18)	0.69
Relative amplitude	0.9 (0.9, 0.9)	0.8 (0.7, 0.9)	0.15
RMSSD [c]	75.6 (66.6, 109.9)	60.4 (50.1, 162.4)	0.56
RMSSD/SD [d]	1.2 (1.1, 1.3)	1.1 (1.1, 1.2)	0.39
Number of immobile minutes daytime	650 (529.5, 686)	601.0 (429.5, 648.7)	0.35
Number of immobile minutes nighttime	673 (544.5, 690.5)	542 (523.5, 644.5)	0.20

[a] Activity intensity of 10-hour window with highest sum of activity intensity.
[b] Activity intensity of 5-hour window with lowest sum of activity intensity.
[c] Root Mean Square of Sequential Differences.
[d] Root Mean Square of Sequential Differences/Standard Deviation.

Table S5. Movement features for the ankle, comparing between the delirious and non-delirious groups.

Sources at 1m	Sound Pressure	Sound Pressure Level (reference sound pressure = 0 dB)
Threshold of pain	20 Pa	120 dB
Pneumatic hammer	2 Pa	100 dB
Street traffic	0.2 Pa	80 dB
Talking	0.02 Pa	60 dB
Library	0.002 Pa	40 dB
TV studio	0.0002 Pa	20 dB
Threshold of hearing	0.00002 Pa	0 dB

Table S6. Examples of sound pressure and sound pressure levels.

References

1. Wang, H., Liang, X., Zhang, H., Yeung, D.-Y. & Xing, E. P. Zm-net: Real-time zero-shot image manipulation network. *arXiv preprint arXiv:1703.07255* (2017).
2. Felzenszwalb, P. F., Girshick, R. B., McAllester, D. & Ramanan, D. Object detection with discriminatively trained part-based models. *IEEE Transactions on Pattern Analysis Mach. Intell.* **32**, 1627–1645, DOI: 10.1109/TPAMI.2009.167 (2010).
3. Weinberger, K. Q. & Saul, L. K. Distance metric learning for large margin nearest neighbor classification. *J. Mach. Learn. Res.* **10**, 207–244 (2009).
4. Szegedy, C., Ioffe, S., Vanhoucke, V. & Alemi, A. A. Inception-v4, inception-resnet and the impact of residual connections on learning. In *AAAI*, 4278–4284.
5. Huang, G. B., Ramesh, M., Berg, T. & Learned-Miller, E. Labeled faces in the wild: A database for studying face recognition in unconstrained environments. Report, Technical Report 07-49, University of Massachusetts, Amherst (2007).
6. Cao, Z., Simon, T., Wei, S.-E. & Sheikh, Y. Realtime multi-person 2d pose estimation using part affinity fields. *Comput. Res. Repos.* (2016).
7. Felzenszwalb, P. F. & Huttenlocher, D. P. Pictorial structures for object recognition. *Int. journal computer vision* **61**, 55–79 (2005).
8. Papandreou, G. *et al.* Towards accurate multi-person pose estimation in the wild. *arXiv preprint arXiv:1701.01779* (2017).
9. Sun, M. & Savarese, S. Articulated part-based model for joint object detection and pose estimation. In *Computer Vision (ICCV), 2011 IEEE International Conference on*, 723–730 (IEEE).
10. Long, J., Shelhamer, E. & Darrell, T. Fully convolutional networks for semantic segmentation. In *Proceedings of the IEEE conference on computer vision and pattern recognition*, 3431–3440.
11. Simonyan, K. & Zisserman, A. Very deep convolutional networks for large-scale image recognition. *arXiv preprint arXiv:1409.1556* (2014).
12. Pishchulin, L., Jain, A., Andriluka, M., Thormählen, T. & Schiele, B. Articulated people detection and pose estimation: Reshaping the future. In *2012 IEEE Conference on Computer Vision and Pattern Recognition*, 3178–3185, DOI: 10.1109/CVPR.2012.6248052.
13. Troyanskaya, O. *et al.* Missing value estimation methods for dna microarrays. *Bioinformatics* **17**, 520–5 (2001).
